# *Triticum vulgare* Extract and Polyhexanide (Fitostimoline^®^ Hydrogel/Fitostimoline^®^ Plus Gauze) versus Saline Gauze Dressing in Patients with Diabetic Foot Ulcers: Results of a Randomized Controlled Trial

**DOI:** 10.3390/jcm12103596

**Published:** 2023-05-22

**Authors:** Giuseppe Della Pepa, Gianluca Lombardi, Salvatore Gianfrancesco, Roberto Piccolo, Giovanni Chirico, Micaela Pellegrino, Luigi Santella, Nicola Tecce, Anastasia Volpicelli, Elena Sollo, Lutgarda Bozzetto, Maria Masulli, Gabriele Riccardi, Angela Albarosa Rivellese, Gennaro Saldalamacchia

**Affiliations:** 1 Department of Clinical Medicine and Surgery, Federico II University, 80131 Naples, Italy; lombardi.gian@gmail.com (G.L.); salvo.gianfrancesco@gmail.com (S.G.); robertopic08@gmail.com (R.P.); giovannichirico@outlook.it (G.C.); micaela-94@libero.it (M.P.); luigisantella01@gmail.com (L.S.); teccenicola@gmail.com (N.T.); anastasiavolpicelli@yahoo.it (A.V.); frasamo2006@libero.it (E.S.); lutgarda.bozzetto@unina.it (L.B.); maria.masulli@unina.it (M.M.); riccardi@unina.it (G.R.); rivelles@unina.it (A.A.R.); gsaldala@libero.it (G.S.); 2 Cardiometabolic Risk Unit, Institute of Clinical Physiology, National Research Council—CNR, 56100 Pisa, Italy

**Keywords:** diabetic foot ulcers, dressing, *Triticum vulgare* extract, Fitostimoline^®^ hydrogel, Fitostimoline^®^ Plus gauze, saline gauze

## Abstract

Background: The use of dressings is an essential component of the standard of care for diabetic foot ulcers (DFUs); however, despite the wide variety of dressings available, there is a lack of evidence from head-to-head randomized controlled trials. We evaluated the efficacy and safety of *Triticum vulgare* extract and polyhexanide (Fitostimoline^®^ hydrogel/Fitostimoline^®^ Plus gauze) versus saline gauze dressings in patients with DFUs. Methods: This study involved a monocentric, two-arm, open-label, controlled trial in patients with DFUs (Grades I or II, Stage A or C, based on the Texas classification) randomized to 12 weeks of dressing with Fitostimoline^®^ hydrogel/Fitostimoline^®^ Plus gauze or saline gauze. The number of patients with complete healing, the reduction in DFU size, and the presence of local signs and symptoms of the wound and perilesional skin were evaluated every two weeks and at the end of treatment. Results: A total of 40 adult patients were recruited (20 patients in each treatment group). The proportion of patients with complete healing was similar between the two groups (61% vs. 74%, *p* = 0.495, Fitostimoline^®^ hydrogel/Fitostimoline^®^ Plus gauze vs. saline gauze, respectively), without significant differences, as well as the reduction in DFU size. A significant improvement in local signs and symptoms of the wound and signs of perilesional skin in the Fitostimoline^®^ hydrogel/Fitostimoline^®^ Plus gauze compared with the saline gauze group was observed. Conclusions: In a clinical setting, the use of Fitostimoline^®^ hydrogel/Fitostimoline^®^ Plus gauze dressing in patients with DFUs significantly improves signs and symptoms of the wound and signs of perilesional skin compared with saline gauze dressing with a similar efficacy in terms of wound healing.

## 1. Introduction

Diabetes Mellitus (DM) is one of the most widespread metabolic diseases, and the alarming rise in its prevalence worldwide poses enormous challenges [1]. The microvascular and macrovascular complications of DM heavily impact on longevity and quality of life [1]. Diabetic foot ulcers (DFUs) are among the ten top causes of worldwide disease burden and disability [2], representing the most serious and costly complication of DM. The etiology of DFUs might be neuropathic, ischemic, or neuroischemic [3]. DFU encompasses a wide range of diseases based on acute/chronic insurgence and the clinical severity of the wound [3], which should be characterized according to the etiology, location, size, depth, and concomitant presence of infection [3]. A DFU will be developed in 19–34% of patients with DM during their life [4], and it is estimated that a minor or major lower-extremity amputation is required in approximately 20% of patients with DM developing a DFU [4]. Consequently, it is of paramount importance to carry out early prevention and treatment of DFUs.

Essential components of the care, management, and treatment of DFUs are represented by health education, strict control of blood glucose and cardiovascular risk factors, offloading, local debridement, and adequate dressing [5]. In the last decades, it has been recognized that the use of a dressing has not only the simple function of covering the wound but is also able to promote wound healing [5]. A wide variety of dressings are available, ranging from basic contact dressings (low adherence dressings such as saline gauze, paraffin gauze, or simple absorbent dressings) to advanced dressings (alginate, hydrogel, films, hydrocolloid, foam) [6]. The ideal dressing should be able to promote wound healing, provide protection for the wound, and reduce associated symptoms such as pain, burning, and itching.

Due to a lack of evidence from head-to-head randomized controlled trials (RCTs), the relative effects of any of the dressings available on DFUs remain unclear. In this regard, an evidence-based systematic review and meta-analysis suggests that the use of hydrogel dressings significantly decreased the time of wound healing, resulting in a higher reepithelization proportion rate and a satisfying relief of pain compared with non-hydrogel dressings [7]. Notably, of 29 RCTs and 14 controlled clinical trials, only 12 were performed on patients with DFUs, and among them, only 6 were conducted in the last ten years [7]. Contrarily, a very recent and comprehensive meta-analysis [8] on 36 RCTs performed only in patients with DFUs concludes that hyaluronic acid dressing, amniotic membrane dressing, honey dressing, and platelet-rich plasma dressing are the ideal materials for topical treatment of DFUs compared with conventional dressing, among which saline gauze was included [8]. It is important to underline the limitations of the reported data, represented by the poor quality of many of the trials, the diversity of the debriding agents being compared, the small sample sizes for some trials, and the lack of replication studies. So far, clinical evidence supporting the choice of dressing has been based mostly on the clinician’s perception rather than high-quality evidence.

Among the most common and less expensive dressings in clinical practice for the management of DFUs in outpatient clinics, particularly in less severe forms, there are both advanced contact dressings, such as *Triticum vulgare* extract combined with polyhexanide and poly/oligosaccharide components (Fitostimoline^®^ in different formulations such as gauze, spray, hydrogel, and cream), or basic contact dressings, such as gauzes [9].

The main components of Fitostimoline^®^ are *Triticum vulgare* extract, a specific aqueous germinated wheat extract obtained using a complex and specific process as already described [10], and polyhexanide, a synthetic polymer structurally similar to the naturally occurring antimicrobial peptides [11].

Fitostimoline^®^ is able to favor wound healing with different mechanisms involved in tissue repair, such as the stimulation of chemotaxis, maturation of fibroblasts, granulation tissues, and autolytic debridement [12].

Gauze dressings promote wound healing by favoring superficial debridement, mechanical removal of necrotic tissues, and protection from contamination [13]. The gauze’s functionality might be improved by adding saline [13].

Here, we evaluated the efficacy and safety of *Triticum vulgare* extract and polyhexanide (Fitostimoline^®^ in the form of the medical device Fitostimoline^®^ hydrogel and Fitostimoline^®^ Plus gauze, Farmaceutici Damor SpA, Naples, Italy) versus saline gauze dressing in patients with DFUs in a monocentric, two-arm, open-label, randomized, controlled trial.

## 2. Materials and Methods

### 2.1. Study Design and Participants

We performed a monocentric, two-arm, open-label, randomized, controlled trial in patients with DFUs regularly attending the Diabetic Foot Unit outpatient clinic of the Federico II University Teaching Hospital. This study was performed in order to assess the safety and efficacy of two approved dressings in clinical practice in relation to the recovery rate of wounds after 12 weeks: (1) Fitostimoline^®^ hydrogel and Fitostimoline^®^ Plus gauze and (2) saline gauze dressing. The inclusion criteria were adult patients with DFUs at Grades I or II and at Stage A or C, based on the Texas classification [14], for a period of at least 12 weeks without infection; an ankle brachial index > 0.8; patients able to understand simple instructions; and patients who provided voluntary, signed informed consent. Exclusion criteria were active infection, evidence of ischaemia in the limb, osteomyelitis, gangrene, systemic inflammatory or autoimmune disease, use of corticosteroids, immunosuppressive agents, radiation therapy and chemotherapy, and known hypersensitivity to any of the dressing components.

The protocol and informed consent were reviewed and approved by the Federico II Ethic Committee and this study was registered at ClinicalTrials.gov (NCT05661474). This study was performed in compliance with the Declaration of Helsinki and the Guidelines for Good Clinical Practice. The patients provided written informed consent for both treatments and then underwent screening to determine eligibility for this study according to the inclusion and exclusion criteria.

Patients were randomized for either Fitostimoline^®^ hydrogel and Fitostimoline^®^ Plus gauze (Fitostimoline^®^ hydrogel/Fitostimoline^®^ Plus gauze group) or saline gauze (saline gauze group) dressing local wound care by a computer randomization program (MINIM software, http://www.users.york.ac.uk access on 6 April 2023). Minimization was performed to stratify for sex, age, underlying etiology (neuropathic, ischemic, or neuroischemic), and size of the wound.

Fitostimoline^®^ hydrogel is a medical device consisting of a gel contained in an aluminum tube. The qualitative composition includes mainly *Triticum vulgare* extract—i.e., Rigenase^®^—and polyhexanide combined with glycerine, macrogol 400, phenoxyethanol, hydroxyethyl cellulose, and purified water [15].

Fitostimoline^®^ Plus gauze is a medical device consisting of single-use gauze dressings impregnated with water-dispersible cream. The qualitative composition includes *Triticum vulgare* extract—i.e., Rigenase^®^—polyhexanide, polyethylene glycol 400, polyethylene glycol 600, polyethylene glycol 1500, polyethylene glycol 4000, glycerine, phenoxyethanol, and purified water [15].

After the enrolment, at visit (V) 1, all patients were assessed based on regular physical examination and general laboratory tests (complete blood count, routine chemistry screen, urinary analysis, and vital signs check-up), and their previous medical records were reviewed. Next, the wounds of all the patients included in this study underwent sharp surgical debridement to remove necrotic tissue and slough. After debridement operations, disinfection with povidone-iodine, and cleansing with sterile saline solution, the wounds were treated as follows: for treatment (1), Fitostimoline^®^ hydrogel was applied and covered with Fitostimoline^®^ Plus gauze; for treatment (2), saline gauze was applied; and finally, in both treatments a layer of sterile gauze was applied over the medication to completely cover the wound area. All the procedures were conducted by the investigators at each V of control. At V1, the patients were instructed to repeat the assigned medication every day at home, and instructions at each V were reinforced.

The wound sizes (the smallest and the largest diameter, depth, and area evaluated as the product of the two longest perpendicular dimensions) and the presence of local signs and symptoms of the wound and perilesional skin were recorded at baseline (V1) and thereafter (i.e., on V2, V3, V4, V5, V6, and V7). The wound sizes were measured using a centimeter ruler, and the presence of local signs and symptoms of the wound and perilesional skin was evaluated using a questionnaire for which for each sign or symptom there was a scale: 0 (absence), 1 (moderate), 2 (mild), and 3 (severe). In particular, the local signs of the wound were represented by erythema and bleeding, while the symptoms were represented by pain, burning, and itching. The local signs of perilesional skin were represented by erythema, oedema, and dry and flaky skin, while the symptoms were represented by pain, burning, and itching. The score ranged from 0 to 6 for the signs of the wound and from 0 to 9 for the symptoms of the wound and the signs/symptoms of perilesional skin, according to the sum of the entity of each sign or symptom. The local signs of the wound and perilesional skin were evaluated by the same physician, while the questionnaire on the local symptoms was scored by the patients during each visit after each dressing change.

All the described measurements were recorded at baseline (V1) and thereafter (i.e., on V2, V3, V4, V5, V6, and V7). Any untoward side effects were recorded every two weeks with examinations on V1, V2, V3, V4, V5, V6, and V7. A follow-up evaluation was conducted every 2 weeks.

The primary outcome was the proportion of patients who, at the end of this study period of 12 weeks (V7), were categorized as complete responders—complete healing of the wound defined as reepithelialisation of 100% without medications. The secondary outcomes included the time to complete reepithelialisation from V1 at any interval; the proportion of patients categorized as partial responders (50% or greater reduction in the product of the two longest perpendicular diameters from baseline); the reduction of the area of the wound in patients categorized as non-complete responders (less than 50% reduction in the product of the two longest perpendicular diameters from baseline); the evaluation of local signs and symptoms of the wound and perilesional skin; and the safety and tolerability of treatments.

Primary and secondary outcomes were detected at V2: 2 weeks; V3: 4 weeks; V4: 6 weeks; V5: 8 weeks; V6: 10 weeks; and V7: 12 weeks.

### 2.2. Other Outcomes

Body weight, height, and waist circumference were measured using standard procedures, and body mass index (BMI) was calculated as weight (kg)/height (m^2^). Blood samples were obtained in the morning after an overnight fast, and all biochemical analyses were performed at the outpatient laboratory of the Clinical Hospital Laboratory of Federico II University of Naples, using standard procedures. Total and HDL cholesterol were measured using standard methods. LDL cholesterol was calculated according to the Friedewald equation only for triglyceride values < 400 mg/dL. Glycated hemoglobin (HbA1c) was measured using high-performance liquid chromatography standardized according to the IFCC.

### 2.3. Sample Size

To be able to discern a clinically relevant difference in the success rate greater than 50% in the treatment with Fitostimoline^®^ hydrogel/Fitostimoline^®^ Plus gauze compared to the saline gauze dressing, with an 80% power at a 5% significance level, considering a drop-out rate of 20%, a total of 40 patients were recruited, 20 for each treatment group.

### 2.4. Statistical Analysis

Continuous variables were expressed as mean ± standard deviation. Categorical variables were described as counts and percentages. Within groups, before–after intervention differences were evaluated using a paired sample *t*-test. The χ^2^ test was used to examine the association between the groups and categorical variables. Between-treatment differences were evaluated using a ANCOVA general linear model taking variable changes (12 weeks minus baseline) as dependent variables and treatment as a fixed factor. For all analyses, the level of statistical significance was set at *p* < 0.05. Statistical analysis was conducted using the SPSS Statistics software 28.0 (SPSS/PC; IBM, Armonk, NY, USA).

## 3. Results

### 3.1. Baseline Characteristics of the Participants

**Figure 1:**
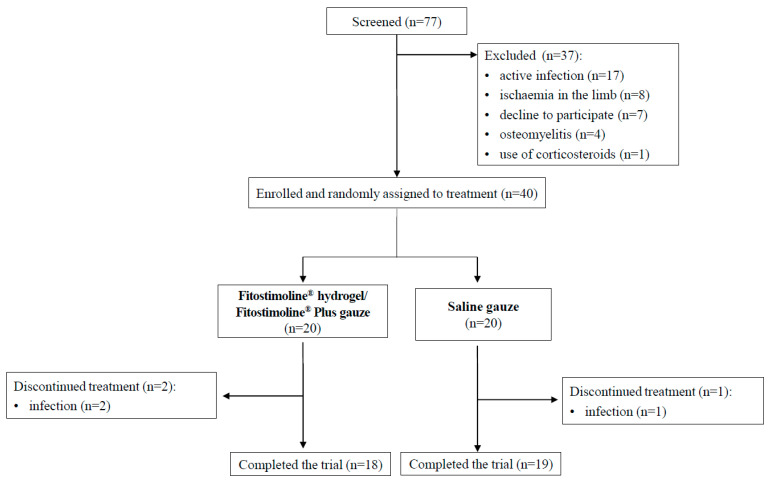
Flowchart of the study.

From 1 February 2021 to 12 July 2022, 77 patients were screened for eligibility, and 40 were randomized (Figure 1). Thirty-seven patients (eighteen in the Fitostimoline^®^ hydrogel/Fitostimoline^®^ Plus gauze group and nineteen in the saline gauze group) completed this study, while three participants (two in the Fitostimoline^®^ hydrogel/Fitostimoline^®^ Plus gauze group and one in the saline gauze group) were drop-outs due to the development of a concomitant infection of the wound during this study.

Anthropometrics, clinical and biochemical parameters, and wound characteristics were similar between groups at baseline (Table 1). On average, patients were 74% male, equally distributed among groups, with an average age of 64 years and a body mass index of 30.5 kg/m^2^. Blood glucose control and fasting plasma lipids were similar between groups (Table 1). The average duration of the wounds was 6 months, and the etiology was mostly neuropathic.

The sizes and areas of the wounds were comparable between groups (Table 1).

### 3.2. Effects of the Two Treatments on the Wound Sizes

**Figure 2:**
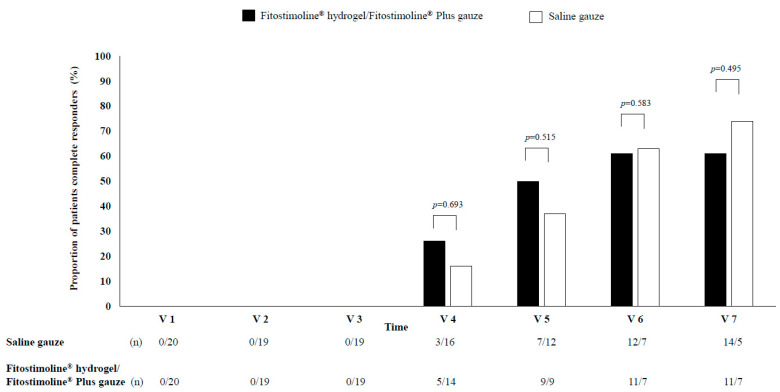
Proportion of complete responders (patients showing complete healing of the wound) in the two treatments from visit four to the end of this study. V: visit, n: number of patients. The bars represent the proportion of patients with complete healing of the wound. For each visit, the number of patients with complete healing of the wound is reported, followed by the number of patients without complete healing of the wound.

After 12 weeks of treatment (V7), 11 patients in the Fitostimoline^®^ hydrogel/Fitostimoline^®^ Plus gauze group and 14 patients in the saline gauze group had complete healing of the wound (complete responders). The proportion of complete responders was similar between the two groups (61% vs. 74%, *p* = 0.495, Fitostimoline^®^ hydrogel/Fitostimoline^®^ Plus gauze vs. saline gauze, respectively), without significant differences (Figure 2). The proportion of complete responders was also evaluated at each visit, and no significant differences were observed between treatments. In detail, complete responders were observed starting from V4 (26% vs. 16%, *p* = 0.693 Fitostimoline^®^ hydrogel/Fitostimoline^®^ Plus gauze vs. saline gauze, respectively) and increased progressively from V5 (50% vs. 37%, *p* = 0.515 Fitostimoline^®^ hydrogel/Fitostimoline^®^ Plus gauze vs. saline gauze, respectively) to V6 (61% vs. 63%, *p* = 0.583 Fitostimoline^®^ hydrogel/Fitostimoline^®^ Plus gauze vs. saline gauze, respectively) (Figure 2).

At V7, seven patients in the Fitostimoline^®^ Plus group and five patients in the saline gauze group did not show complete healing of the wound (partial or non-complete responders). The proportion of partial responders was similar between groups (38% vs. 21%, *p* = 0.471, Fitostimoline^®^ hydrogel/Fitostimoline^®^ Plus gauze vs. saline gauze, respectively), without significant differences. One patient in the saline gauze group was categorized as a non-complete responder.

In non-complete and partial responders (39% vs. 26%, Fitostimoline^®^ hydrogel/Fitostimoline^®^ Plus gauze vs. saline gauze, respectively), there was a significant reduction in the largest and smallest diameters, depth, and area of the wounds for both groups without any significant difference between groups (Table 2).

No adverse events were reported in relation to the procedures or to the products during the interventions.

### 3.3. Effects of the Two Treatments on Signs and Symptoms of the Wound and Perilesional Skin

**Figure 3:**
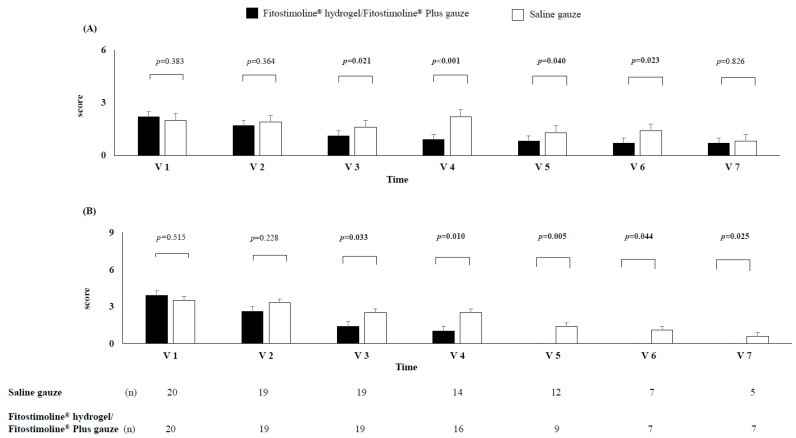
Effects of the two treatments on the score of the local signs (**A**) and symptoms (**B**) of the wound at each visit. V: visit, n: number of patients. The bars represent the score for local signs and symptoms of the wound. The local signs of the wound were represented by erythema and bleeding, while the symptoms were represented by pain, burning, and itching. For each visit the number of patients without complete healing of the wound is reported.

From V3 to V6, a significant reduction in the score of erythema and bleeding of the wound was observed in the Fitostimoline^®^ hydrogel/Fitostimoline^®^ Plus gauze group compared with the saline gauze group (Figure 3A). Similarly, from V3 to V4, a significant reduction in the score of pain, burning, and itching of the wound was observed in the Fitostimoline^®^ hydrogel/Fitostimoline^®^ Plus gauze group compared with the saline gauze group, with a complete absence of these symptoms from V5 to V7 only in the Fitostimoline^®^ hydrogel/Fitostimoline^®^ Plus gauze group (Figure 3B).

**Figure 4:**
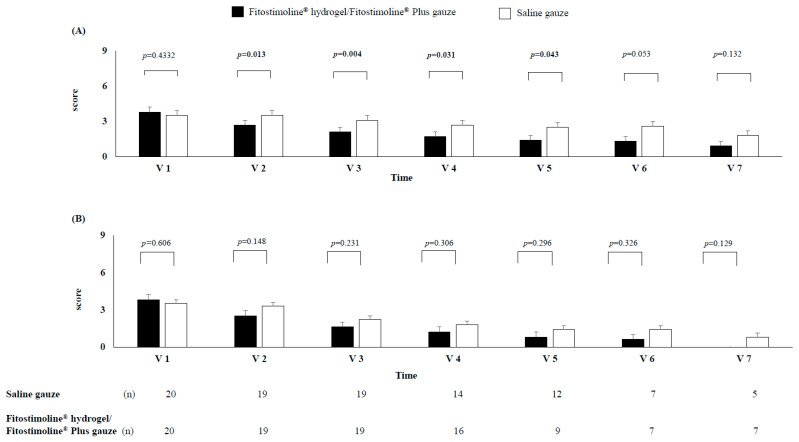
Effects of the two treatments on the score of the local signs (**A**) and symptoms (**B**) of perilesional skin at each visit. V: visit, n: number of patients. The bars represent the score for local signs and symptoms of perilesional skin. The local signs of perilesional skin were represented by erythema, oedema, and dry and flaky skin, while the symptoms were represented by pain, burning, and itching. For each visit, the number of patients without complete healing of the wound is reported.

With regard to signs and symptoms of perilesional skin, from V2 to V5 a significant reduction in the score of erythema, oedema, and dry and flaky skin was observed in the Fitostimoline^®^ hydrogel/Fitostimoline^®^ Plus gauze group compared with the saline gauze group (Figure 4A), while the symptoms of pain, burning, and itching similarly decreased among the groups (Figure 4B).

## 4. Discussion

In our study, we have shown that in a clinical setting, the use of Fitostimoline^®^ hydrogel/Fitostimoline^®^ Plus gauze dressing in patients with DFUs compared with saline gauze dressing (1) has a similar efficacy in terms of complete/partial wound healing and (2) significantly improves the signs and symptoms of the wound and the signs of perilesional skin.

The first major finding of our study was the comparable rate of wound healing in both treatment groups, as well as the reduction in the largest, smallest, depth, and area of the wounds at each visit measurement, without significant differences between the two groups.

Current evidence shows that a wide variety of dressings are available for the treatment of DFUs; on the other hand, data from head-to-head RCTs are lacking, and the relative effects of any of these dressings in DFUs remain unclear. Recent meta-analyses report that among different dressings, the application of hydrogel dressings, as well as other forms of dressing such as amniotic membrane, honey, and platelet-rich plasma dressings, can significantly shorten the healing time of DFUs and can also effectively improve the cure rate of DFUs compared with other conventional dressings, which include saline gauze [7,8].

Unfortunately, some of the performed trials had a small and underpowered sample size; furthermore, the lack of baseline comparability, different times of intervention, the diversity of the debriding agents being compared, and the lack of replication studies should be considered [7,8]. In our trial, the two-arm, open-label, randomized, controlled design, the 12 weeks of treatment sufficiently adequate for the evaluation of wound healing, and the similar characteristics at baseline of the participants, without differences in terms of risk factors for DFUs, encompasses some limitations of the previous trials. Moreover, the equal effects of the two treatments in our trial might be due to the tight management of diabetes and the other cardiovascular risk factors in all patients during each visit according to the clinical practice of a tertiary clinical setting. In addition, even the treatment with the saline gauze dressing was implemented with particular care, surely superior to that generally used in clinical practice. In fact, the proportion of complete healing was considerably high with both treatments (more than 60%), and even more clinically relevant was the proportion of complete and partial healing, 95% for saline gauze and 100% for Fitostimoline^®^ hydrogel/Fitostimoline^®^ Plus gauze.

Fitostimoline^®^ is widely used for the treatment of different lesions, including DFUs, and its safety and efficacy have been recognized for decades. The active compound of Fitostimoline^®^—Rigenase^®^ based on the aqueous extract of *Triticum vulgare*, combined with polyhexanide, which prevents colonization and contamination of the wound [12]—favors the different mechanisms involved in tissue repair, such as the stimulation of chemotaxis, maturation of fibroblasts, granulation tissues, and autolytic debridement, improving the healing processes [12]; furthermore, the high water content of hydrogel is able to retain oxygen, promote exudate absorption of the ulcer, and favor the optimal *milieu* to promote the physiological healing processes of the wound [13,16,17,18].

Saline gauze is still commonly used for DFU dressing and is less cost-effective. Despite its widespread use, the plausible mechanisms of action are not yet understood, and they might be related to the mechanical debridement that occurs with daily dressing changes, which might promote wound healing [19,20].

The second finding of our study was the significant improvement in signs and symptoms related to DFUs. In particular, Fitostimoline^®^ hydrogel/Fitostimoline^®^ Plus gauze compared with saline gauze significantly decreased the score of erythema, bleeding, pain, burning, and itching at the level of the wound, together with an improvement in erythema, oedema, and dry and flaky skin of the perilesional skin.

Our data are in accordance with evidence [7] indicating that hydrogel dressings can effectively alleviate the pain and burning and irritating sensations typical of skin wounds, and data obtained from in vitro studies have shown that *Triticum vulgare* extract reduces the production of nitric oxide, Interleukin-6, Prostaglandin E2, and Tumor Necrosis Factor α [14] involved in the signs and symptoms associated with inflammation.

Oxidative stress, inflammation, and dry and flaky skin play a remarkable role in the development of local signs and symptoms; consequently, the antioxidant activity of Fitostimoline^®^ might contribute to these findings [21] also by promoting the protection of the terminals of the peripheral nerve [22].

These effects offer greater patient comfort and compliance. On the other hand, the minimal reduction in the score of local signs and symptoms in the saline gauze groups might be related to the properties of gauze, which can become moistened and tends to become adherent to the wound, promoting pain during its removal. From a clinical point of view, the discomfort caused by the changing of saline gauze dressings at each visit might reduce patients’ compliance and quality of life, promoting financial and psychological costs.

In our study, the confirmed safety results showed that both treatments were safe and well tolerated in terms of local and general adverse effects.

Our study presents some limitations to be considered. First, only patients with DFUs at Grades I or II, and at Stage A or C, according to the Texas classification, were recruited; consequently, the results cannot be generalized to all DFUs with severe ischemia or severe infection, in which the concomitant treatment with antibiotics could lead to possibly different results. Second, despite the randomized and controlled design, the wound measurements were not taken in a blinding condition, and the open-label application of the two dressings represents a further limitation. Third, the evaluation of signs and symptoms of the wound and perilesional skin was measured using a score in which some domains were subjective. Fourth, the small sample size might represent a further limitation. Finally, a longer trial duration might allow for the observation of a higher proportion of complete wound healing.

Therefore, further clinical trials with a longer duration, an adequate sample size, and which also take into account the comparison between different hydrogels are needed.

## 5. Conclusions

In conclusion, we found a similar good efficacy of Fitostimoline^®^ hydrogel/Fitostimoline^®^ Plus gauze and the saline gauze dressings for local DFU care regarding the proportion of complete wound healing in a clinical setting; however, a significant improvement in terms of local signs and symptoms of the wound and the signs of perilesional skin was observed in the Fitostimoline^®^ hydrogel/Fitostimoline^®^ Plus gauze group, suggesting more patient comfort during the weeks of treatment. The results of this randomized trial might aid clinicians in the choice of a wide variety of dressings, taking into consideration patient discomfort.

## Figures and Tables

**Table 1:** Baseline characteristics of the participants.

	Fitostimoline^®^ Hydrogel/ Fitostimoline^®^ Plus Gauze (*n* = 20)	Saline Gauze (*n* = 20)
Sex (female/male)	4/16	7/13
Age (years)	65 ± 10	63 ± 9
BMI (kg/m^2^)	31 ± 5	30 ± 5
HbA1c (%)	7.7 ± 1.3	7.4 ± 0.7
Total Cholesterol (mg/dL)	157 ± 37	143 ± 29
HDL Cholesterol (mg/dL)	43 ± 11	45 ± 9
Triglycerides (mg/dL)	131 ± 61	124 ± 53
LDL Cholesterol (mg/dL)	89 ± 37	74 ± 34
Wound duration (months)		
	6 ± 6	7 ± 6
Wound etiology		
Neuropathic	8	9
Ischemic	7	6
Neuroischemic	5	5
Wound size		
Wound largest diameter (cm)	1.7 ± 0.9	1.6 ± 1
Wound smallest diameter (cm)	1.1 ± 0.5	1.1 ± 0.8
Wound depth (cm)	0.4 ± 0.2	0.4 ± 0.2
Wound area (cm^2^)	2.1 ± 1.8	2.3 ± 2.7

Data are expressed as mean ± standard deviation or number. BMI, body mass index; HbA1c, glycated hemoglobin. No differences at baseline between groups.

**Table 2:** Wound size reduction in partial/non-complete responders (patients without complete healing of the wound) after 12 weeks of treatment.

	Fitostimoline^®^ Hydrogel/ Fitostimoline^®^ Plus Gauze (*n* = 7)			Saline Gauze (*n* = 5)			p ^†^
Wound Size	Baseline	12 Weeks	Δ	Baseline	12 Weeks	Δ	
Wound largest diameter (cm)	2.2 ± 1	0.8 ± 0.6 *	−1.4 ± 0.6	2.8 ± 1.1	1.4 ± 0.9 *	−1.5 ± 0.5	0.831
Wound smallest diameter (cm)	1.4 ± 0.6	0.4 ± 0.2 *	−1.0 ± 0.5	1.9 ± 0.8	0.8 ± 0.9 *	−1.0 ± 0.5	0.992
Wound depth (cm)	0.5 ± 0.2	0.3 ± 0.2 *	−0.3 ± 0.2	0.7 ± 0.3	0.5 ± 0.4 *	−0.3 ± 0.2	0.697
Wound area (cm^2^)	3.2 ± 1.9	0.3 ± 0.2 *	−2.7 ± 1.7	5.5 ± 3.0	1.6 ± 2.0 *	−3.9 ± 1.9	0.300

Data are expressed as mean ± standard deviation. * *p* < 0.05 vs. baseline. ^†^ *p* for between-treatment differences in variable changes (12 weeks minus baseline).

## Data Availability

The data that support the findings of this study are available from the corresponding author upon reasonable request.
